# Depression is an independent risk factor for stroke reccurence and cognitive impairment in stroke patients

**DOI:** 10.1038/s41398-025-03706-8

**Published:** 2025-11-21

**Authors:** Seyda Cankaya, Shair Shah Safa, Ayse Karakus, Mehmet Savcili, Lutfu Hanoglu, Adil Mardinoglu, Fatma Ece Cetin, Emre Kumral, Burak Yulug

**Affiliations:** 1 Department of Neurology and Neuroscience, Alanya Alaaddin Keykubat University, Faculty of Medicine, Antalya, 07425 Türkiye; 2 Department of Neurology and Neuroscience, Istanbul Medipol University, Faculty of Medicine, Istanbul, 34214 Türkiye; 3 Centre for Host-Microbiome Interactions, Faculty of Dentistry, Oral & Craniofacial Sciences, King’s College London, London, SE1 9RT United Kingdom; 4 Science for Life Laboratory, KTH-Royal Institute of Technology, Stockholm, SE-171 21 Sweden; 5 Acıbadem Hospital, Department of Neurology, Bursa, Türkiye; 6 Ege University, Faculty of Medicine, Department of Neurology, Izmir, Türkiye

**Keywords:** Depression, Human behaviour

## Abstract

Post-stroke depression (PSD) is a significant sequela of cerebrovascular accidents, affecting a substantial proportion of stroke survivors. However, it is still unclear whether the existence of depression after stroke is an independent risk factor for stroke recurrence and if the increased risk of cognitive impairment in PSD is related to the location of stroke. We aimed to compare the role of cortical, subcortical and cortico-subcortical infarcts in the development of PSD and cognitive impairment, as well as the role of the existence of depression in stroke recurrence. In this study, a 52-week, randomised, double-blind study consisted of 1059 stroke patients (866 non-depressive and 193 untreated depressive persons) who were matched in terms of demographic and clinical parameters. The Mini Mental State Examination Test (MMSE), Executive function (Trail Making Test Part A), processing speed (colour naming condition of the Stroop test), episodic memory (Rey Auditory Verbal Learning Test [RAVLT], including delayed free recall), semantic memory (verbal fluency test [animal naming]), language processing (Boston Naming Test [(number correct]), visuospatial perception (the bells test) was assessed at the baseline. The lesion sites are subdivided as cortical, subcortical, and cortico-subcortical territory infarcts on MRI. The stroke recurrence ratio was also recorded after a year. In results, we observed a higher rate of depression associated with lesions affecting the cortico-subcortical structures in patients with PSD compared to non-depressive patients (p < 0.05). Our results further indicated impaired cognitive scores in patients with PSD compared to those with non-depressive individuals (p < 0.05). Regarding the risk of stroke recurrence, we also found an increased rate of stroke recurrence in PSD after 12 months (p < 0.05). In detail, binomial logistic regression analyses using the backward Wald method determined that patients with depression (p = 007; odds ratio (OR) = 1.64; CI 1.14–2.35), hypertension (p = 0.004; OR = 1.74; CI 1.19–2.55), atrial fibrillation (p = 0.007; OR = 1.61; CI 1.14–2.28) and older age (p = 0.019; OR = 1.02; CI 1.003–1.03) were significantly predictors for stroke recurrency. Our regression analysis further revealed that PSD was a predictive factor for disabling cognitive test scores (impaired executive function [p < 0.001; OR = 4.51; CI 3.24–6.27], reduced processing speed [p < 0.001; OR = 4.29; CI 3.12–5.91], episodic memory [p < 0.001; OR = 4.65; CI 3.37–6.42), semantic memory [p < 0.001; OR = 4.79; 3.47–6.61], visuospatial [p < 0.001; OR = 6.10; CI 4.36–8.55], and language function [p < 0.001; OR = 5.086; CI 3.67–7.05]) after adjusting for age and education. In conclusion, the present study provides strong evidence confirming the importance of depression in predicting cognitive impairment and recurrence in stroke patients. Despite these positive findings, our findings warrant the performance of further research to demonstrate the efficacy of treatment on stroke recurrence, together with other vascular risk factors and cognitive disorders.

## Introduction

Post-stroke depression (PSD) is a significant sequela of cerebrovascular accidents, affecting approximately 30 to 65% of stroke survivors, depending on various factors, including the timing of assessments and the population studied [1, 2]. The pathogenesis of PSD is complex and multifactorial, involving interactions among biological, psychological, and social factors, as well as specific neuroanatomical correlates and inflammatory processes. Although several inflammatory cytokines and neurochemical alterations involving neurotransmitter systems such as serotonin and monoamines have been implicated in the pathophysiology of PSD [3, 4], the neuroanatomical correlations between the location of the stroke and the emergence of depressive symptoms remain unclear. For instance, strokes affecting the left hemisphere, particularly in areas such as the frontal lobe, have been linked to higher incidences of depression [5]. In contrast, there are several studies indicating the role of the right hemisphere associated with strategic lesions in the emergence of PSD [6, 7]. However, in addition to these positive results, there are also studies indicating no significant association between PSD and lesion location [8, 9]. These findings together suggest that comprehensive evaluations of regional brain connections, rather than isolated examinations of individual cortical regions, are essential if the neurocognitive mechanisms underlying PSD are to be elucidated. For example, it was observed that while early PSD had various determinants, lesions, including cortical and subcortical atrophy, became more prominent in terms of depressive symptoms over a more extended period post-stroke [10]. It is not unreasonable to assume that strokes involving strategic locations, including cortico-subcortical sites, rather than a pure cortical or subcortical location, may represent a determinant in the pathophysiology of PSD. This assumption aligns with the previous definition of the pathophysiology of depression in terms of an impaired function of the corticolimbic axis (similar to cortico-subcortical locations, as in the present case), as proposed by Mayberg et al. [11].

On the other hand, cognitive impairment is more prominent in PSD [12–14]. This is supported by several studies under the umbrella of the vascular depression hypothesis [4], indicating an increased risk of cognitive impairment in PSD [15]. This may be due to overlapping vascular risk profiles, which have already been identified as risk factors for the development of stroke, cognitive impairment, and dementia [16, 17]. For instance, while depression may be influenced by disruptions in brain circuits related to mood regulation and cognition [18, 19], a greater burden of white matter hyperintensity, a pathophysiological aspect of vascular depression, is associated with cognitive processing speed, executive function, and memory deficits [19]. Although the fact that vascular depression increases the risk of cognitive impairment is unsurprising, this does not explicitly elucidate the mechanistic link between stroke and depression. In other words, despite the positive studies listed above, which suggest that PSD has its underlying biological causes, it is difficult to exclude the bidirectional relationship between depression, stroke recurrence, and cognitive impairment. Interestingly, this might even include a plausible association between PSD and post-stroke cognitive and functional deficits, indirectly suggesting that PSD may constitute a psychological reaction to these deficits. This might be due to numerous psychosocial risk factors for PSD and depression without stroke, such as previous psychiatric history, premorbid neurotic personality traits, and social isolation. However, confirming this will be challenging, and further, specifically designed studies are therefore needed.

To the best of our knowledge, no previous specific, randomised study has compared the role of cortical, subcortical, and cortico-subcortical infarcts in the development of PSD and cognitive impairment, and the role of the existence of depression in stroke recurrence. Due to this gap in the existing literature, we aimed to compare the role of cortical, subcortical, and cortico-subcortical infarcts in the development of PSD and cognitive impairment, as well as the role of the existence of depression in stroke recurrence. Here, we hypothesised that cortico-subcortical lesion location, along with depression, might play a crucial role in the development of post-stroke depression and stroke recurrence.

## Material and methods

Data were obtained from the prior prospective study published by Ece Çetin et al. [20]. The trial was approved by Ege University medical ethics committee in Izmir (reference EUEC 2013/45) and registered on ClinicalTrials.gov (Treatment Outcomes of Depression; identifier: NCT04776226). Written informed consent was obtained from all participants. All methods were carried out in accordance with the Declaration of Helsinki and relevant institutional and national guidelines and regulations.

### Study design

Eligible patients were adults (aged ≥ 50 years) with a clinical diagnosis of acute stroke within the previous month, brain imaging consistent with ischemic stroke, and a persisting neurological deficit at the time of randomisation. We have selected the patients without depression and those with depressive symptoms without antidepressant treatment among the stroke patients from the database. Post-stroke depression was identified when a major depressive episode developed in the setting of clinical and/or neuroimaging evidence indicative of cerebrovascular disease. Depression was assessed using two approaches: (1) scoring above the established threshold on standardised mood assessment tools, such as the Hamilton Depression Rating Scale (HAM-D) [21], and (2) meeting the diagnostic criteria for either major or minor depression according to the DSM-IV [22] and DSM-5 [23]. The prior study has indicated the inclusion and exclusion criteria [20].

### Neuropsychological measures

The cognitive functions were grouped into rationally selected domains, as described below, based on previous literature regarding the cognitive processes assessed by each task [24–26]. Executive function (Trail Making Test Part A), processing speed (colour naming condition of the Stroop test), episodic memory (Rey Auditory Verbal Learning Test [RAVLT], including delayed free recall), semantic memory (verbal fluency test [animal naming]), language processing (Boston Naming Test [(number correct]), visuospatial perception (the bells test) was assessed at the baseline.

### Neuroimaging criteria

MRI was performed by 1.5 or 3 T scanners (Siemens Sonata, Siemens Medical Solutions, Erlangen, Germany) as part of the standard examination. Routine MRI sequences, including T1-and T2-weighted imaging, DWI, and fluid-attenuated inversion recovery (FLAIR), are obtained. During the imaging process, each T1-weighted structural scan was produced by 190 slices (TR/TE: 8.1/3.7), FOV 256 × 256 × 190 mm (FHxAPxRL), with a voxel size of 1 × 1 × 1 mm which lasted approximately 12 min, leading to the recording of 300 volumes with the following parameters: TR 2230 ms, TE 30 ms, FOV 240 × 240 × 140 mm (RLxAPxFH), voxel size 3 × 3 × 4 mm, flip angle 770, and slice number 35. Finally, we categorised the lesion sites as cortical, subcortical, and cortico-subcortical.

### Statistical analysis

All statistical analyses were conducted using the SPSS 22.0 package for Windows (SPSS Inc., Chicago, IL). Based on a normal distribution, parametric and non-parametric tests were used. Comparisons of continuous variables between groups were conducted using Student’s t-test or the Mann-Whitney U test. The chi-square test was used for categorical variables comparisons, and data were presented as frequencies and percentages. If the test statistic is greater than or equal to 1.96 or less than −1.96, it is significant at the p < 0.05 level in a post-hoc Chi-square test. The One-Way ANOVA test was used for more than two group comparisons. Binomial logistic regression analyses were used to investigate the relationship between depression, baseline neuropsychological functions, risk factors, lesion location and stroke recurrence

## Results

### Demographic, clinical, and cognitive comparisons between groups

A total of 1059 patients were included in the study. 866 non-depressive and 193 depressive stroke patients have been compared with demographic and clinical parameters (Table 1). There were no differences between the groups regarding age, education, and MMSE total score. In terms of gender distribution, 59% (n = 110) of the depression group were male, whereas 51% (n = 343) of the stroke group were male. This difference was statistically significant according to Fisher’s exact test (p = 0.047). In participants, executive function (Trail Making Test Part A), processing speed (colour naming condition of the Stroop test), episodic memory (Rey Auditory Verbal Learning Test [RAVLT], including delayed free recall), semantic memory (verbal fluency test [animal naming]), language processing (Boston Naming Test [(number correct]), visuospatial perception (the bells test) was assessed at the baseline. The mean of all cognitive test scores was significantly lower in PSD than in non-depressive individuals (p < 0.05, Table 1).

**Table 1:** Clinical and demographic characteristics of post-stroke depressive (PSD) patients compared with non-depressive stroke patients.

	PSD (n = 193)		Non-depressive stroke patients (n = 866)		
	Mean (SD)	Median (IQR)	Mean (SD)	Median (IQR)	p
Education (yrs)	7.81 ± 3.169	8.0 (5.0)	8.14 ± 3.18	9.00 (6.00)	0.134
Age	62.69 ± 12.36	64.00 (17.5)	63.80 ± 12.77	65.00 (18.5)	0.261
Gender (male, n, %)	110 (59)		343 (51)		0.047*
Barthel index	65.20 ± 19.34	70.0 (30.0)	64.22 ± 17.95	70.00 (25.0)	0.647
MMSE	25.80 ± 2.385	27.00 (4.0)	25.76 ± 2.97	27.00 (4.00)	0.683
Executive disorder	55.37 ± 13.56	59.00 (28.0)	63.56 ± 10.0	68.0 (12.0)	<0.001*
Reduced processing speed	34.11 ± 5.00	33.00 (6.00)	37.18 ± 4.77	38.0 (2.0)	<0.001*
Episodic memory disorder	4.20 ± 1.40	4.00 (2.50)	5.15 ± 0.98	5.50 (0.60)	<0.001*
Semantic memory disorder	11.16 ± 3.91	11.00 (7.00)	13.65 ± 2.64	14.50 (2.50)	<0.001*
Language processing	41.06 ± 9.13	42.00 (18.0)	46.55 ± 6.41	49.0 (4.00)	<0.001*
Visuospatial skills	26.68 ± 6.24	26.00 (11.0)	31.65 ± 3.90	33.0 (3.00)	<0.001*

Data are presented as mean ± standard deviation (SD), median (interquartile range, IQR), or number (%). Student’s *t*-test and Mann–Whitney U-test were used for continuous variables; chi-square test for categorical variables.

*IQR* inter quantile range; *MMSE* the mini mental statement test; *n* number of patients; *PSD* post-stroke depressive patients.

*p < 0.05 was considered statistically significant.

We also did not find any significance in terms of vascular risk factors such as hypertension (HT), diabetes mellitus (DM), hyperlipidaemia, myocardial infarction history, smoking, obesity, and atrial fibrillation between the groups (Chi-Square test, p > 0.05, Table 2).

**Table 2:** Vascular risk factors and stroke-related parameters in PSD vs non-depressive patients.

		PSD	Non-Depressives	p value
**DM**		50 (26.7)	184 (27.1)	1.00
**HT**		125 (66.8)	483 (71.1)	0.279
**Smoking**		45 (24.1)	201 (29.6)	0.144
**Obesity**		68 (36)	240 (35)	0.796
**MI**		33 (18)	90 (13)	0.155
**Hyperlipidemia**		113 (61)	411 (60.5)	1.00
**Atrial fibrillation**		48 (25.7)	154 (22.7)	0.435
**Stroke Recurrence**		53 (28.3)	128 (18.9)	0.006*
**NIHSS**	**1**	78 (41.7)	267 (39.3)	0.778
	**2**	95 (50.8)	353 (52.0)	
	**3**	14 (7.5)	59 (8.7)	
**Infarct Location**	**Cortical**	70 (37.4)	274 (40.4)	0.032*
	**Subcortical**	63 (33.7)	269 (39.6)	
	**Cortico-subcortical**	54 (28.9)*	136 (20.0)	

Values in parentheses are percentage of each separate column. Categorical variables were compared with Pearson’s χ^2^ or Fisher’s exact test, as appropriate.

*DM* diabetes mellitus; *HT* hypertension; *MI* myocardial infarction; *NIHSS* the national institutes of health stroke scale; *PSD* poststroke depressive patients.

*p < 0.05 was considered statistically significant.

### Group differences in lesion distribution and stroke recurrence

**Fig. 1:**
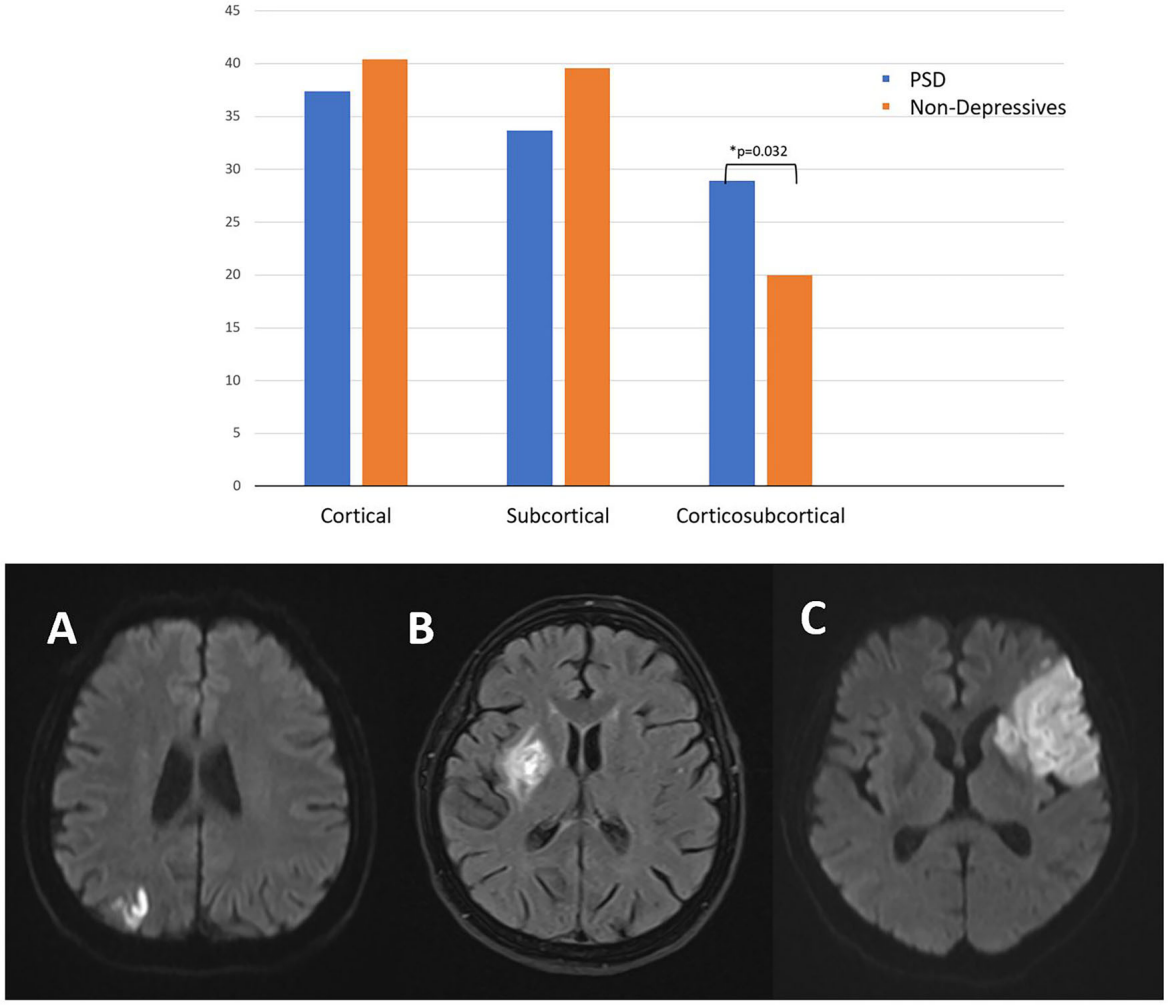
Lesion locations in poststroke depressive (PSD) and non-depressive patients after stroke. The bar graph shows the within-group percentages of cortical, subcortical, and corticosubcortical lesions in PSD (blue) and non-depressive (orange) groups, with a significant difference observed for corticosubcortical lesions (*p = 0.032). Representative axial FLAIR MRI scans illustrate lesion locations: (**A**) Cortical, (**B**) Subcortical, and (**C**) Corticosubcortical.

While infarct locations have been shown to differentiate between the groups (χ²: 6.89, p = 0.032, Table 2), cognitive test scores were the same throughout the locations (One-Way ANOVA, p > 0.05, Table 3). In a post-hoc Chi-Square test, the cortico-subcortical area was most affected in PSD than in non-depressive patients (Table 2, Fig. 1).

**Table 3:** Neurocognitive test scores and demographic characteristics in relation to infarct location among PSD and non-depressive stroke patients.

	Poststroke Depression					Stroke without depression						
	Cortical (n = 70)	Subcortical (n = 63)	Cortico-subcortical (n = 54)	F	p	Cortical (n = 274)	Subcortical (n = 269)	Cortico-subcortical (n = 136)	F	p	F^2^	P^2^
Age	62.84 ± 13.90	64.83 ± 11.67	63.85 ± 12.58	0.398	0.672	62.26 ± 12.02	62.42 ± 13.25	64.07 ± 11.119	1.082	0.339	0.779	0.459
Education	8.54 ± 3.16	7.90 ± 3.18	7.90 ± 3.19	0.877	0.418	7.94 ± 3.11	7.77 ± 3.20	7.59 ± 3.209	0.588	0.556	0.893	0.410
MMSE	26.15 ± 2.39	25.36 ± 2.49	25.72 ± 3.97	1.192	0.306	25.78 ± 2.45	25.7361 ± 2.40511	25.93 ± 2.21	0.314	0.730	0.720	0.487
Executive Disorder	55.5 ± 13.46	54.6 ± 13.73	56.09 ± 13.68	0.179	0.836	63.21 ± 10.40	63.53 ± 9.98	64.3 ± 9.24	0.543	0.581	0.485	0.616
Reduced processing speed	34.31 ± 4.93	33.57 ± 5.32	34.46 ± 4.71	0.556	0.574	36.97 ± 4.54	37.20 ± 5.18	37.5 ± 4.36	0.569	0.566	0.98	0.907
Episodic memory disorder	4.21 ± 1.37	4.01 ± 1.42	4.40 ± 1.39	1.092	0.338	5.14 ± 0.96	5.17 ± 1.01	5.11 ± 1.002	0.209	0.812	0.594	0.553
Semantic memory disorder	10.3 ± 4.10	11.41 ± 3.55	11.99 ± 3.91	3.103	0.12	13.95 ± 2.42	13.44 ± 2.67	13.44 ± 2.94	3.078	0.088	0.894	0.409
Language processing	42.35 ± 8.75	39.52 ± 9.31	41.16 ± 9.26	1.614	0.202	46.10 ± 6.55	46.93 ± 5.90	46.7 ± 7.07	1.196	0.303	0.095	0.909
Visuospatial skills	26.55 ± 6.42	25.46 ± 6.35	28.25 ± 5.58	3.014	0.052	31.69 ± 3.83	31.83 ± 3.73	31.22 ± 4.33	1.136	0.322	0.826	0.438

Values are shown as mean ± SD. One-way ANOVA was used for group comparisons (F, *p*) within groups. Between-group comparisons are reported as F2 and p2.

*MMSE* the mini mental statement test; *n* number of patients; *PSD* post-stroke depressive patients.

Regarding stroke recurrence, patients with depression have a higher recurrence ratio (28.3%) than non-depressive stroke subjects (18.9%) in 52 weeks (χ²= 7.99, p = 0.006).

### Logistic regression analysis

**Fig. 2:**
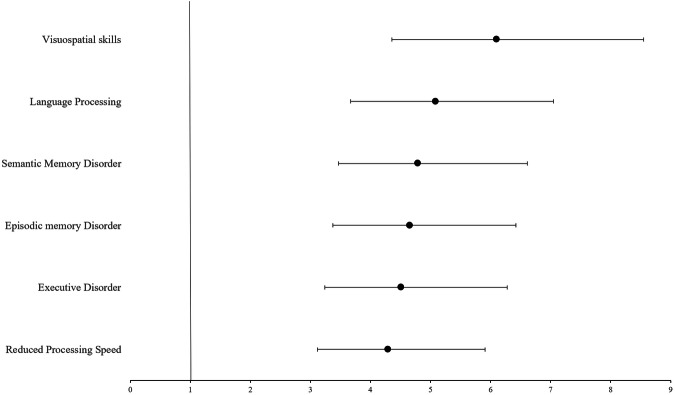
Forest plot of the binary logistic regression model assessing the effect of poststroke depression on different cognitive domains after adjusting for age and education. Dots represent odds ratios (OR) for impairment in each domain, and horizontal lines indicate 95% confidence intervals (CI). The vertical reference line at OR = 1 represents no effect.

In binomial logistic regression analyses, PSD was a predictive factor for disabling cognitive test scores after adjusting for age and education. PSD was significantly predicted impaired executive function (p < 0.001; odds ratio (OR) = 4.51; CI 3.24–6.28), reduced processing speed (p < 0.001; OR = 4.29; CI 3.12–5.91), episodic memory (p < 0.001; OR = 4.65; CI 3.37–6.42), semantic memory (p < 0.001; OR = 4.79; 3.47–6.61), visuospatial (p < 0.001; OR = 6.1; CI 4.36–8.55), and language function (p < 0.001; OR = 5.086; CI 3.67–7.05) in regression analyses (Table 4, Fig. 2).

**Table 4:** Binary logistic regression analysis of the effects of education, age, and diagnosis (PSD vs non-depressive) on impaired neurocognitive test scores.

	Executive Disorder	p	Reduced Processing Speed	p	Episodic memory Disorder	p	Semantic Memory Disorder	p	Language Processing	p	Visuospatial skills	p
Education	1.04 (0.99–1.09)	0.09	1.00 (0.96–1.05)	0.861	0.98 (0.93–1.03)	0.36	1.00 (0.96–1.05)	0.98	1.04 (0.99–1.09)	0.096	1.01 (0.99–1.02)	0.12
Age	1.00 (0.99–1.01)	0.97	0.99 (0.98–1.01)	0.325	1.01 (0.99–1.02)	0.36	1.00 (0.99–1.02)	0.58	1.01 (0.99–1.03)	0.066	1.03 (0.97–1.08)	0.32
PSD vs Non-depressives	4.51 (3.24–6.28)	<0.001*	4.29 (3.12–5.91)	<0.001*	4.65 (3.37–6.42)	<0.001*	4.79 (3.47–6.61)	<0.001*	5.09 (3.67–7.05)	<0.001*	6.10 (4.36–8.55)	<0.001*

Data are presented as odds ratios with 95% confidence intervals (CI).

PSD: Post-stroke depressive patients.

*p < 0.05 indicates statistical significance.

**Fig. 3:**
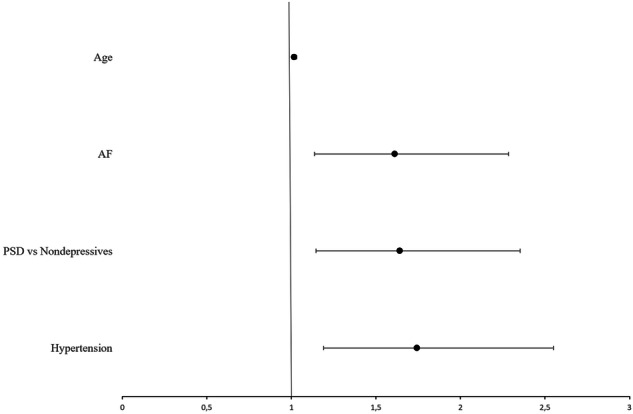
Forest plot of the binary logistic regression model showing the effects of age, atrial fibrillation (AF), poststroke depression (PSD) vs. non-depressive patients after stroke, and hypertension on stroke recurrence. Variables included represent those retained in the model after backward Wald logistic regression. Dots represent odds ratios (OR), and horizontal lines indicate 95% confidence intervals (CI). The vertical reference line at OR = 1 indicates no effect.

Furthermore, binomial logistic regression analyses have determined the predictive factors of stroke recurrence. First, all vascular and demographic risk factors (gender, hypertension, diabetes mellitus, smoking, hyperlipidemia, atrial fibrillation, age, education, the National Institutes of Health Stroke Scale (NIHSS) score, and poststroke depression) were entered individually into univariate logistic regression analyses. In this step, several factors, including depression, hypertension, atrial fibrillation, and older age, showed significant associations with stroke recurrence (Table 5, Fig. 3).

**Table 5:** Binary Logistic Regression Analysis of the Effect of Vascular Factors and Demographical Characteristics on the Dependent Variable Stroke Recurrence.

Variables	Category	N (%)	Univariate OR (%95 CI)	p	Multivariable OR (%95 CI)	p	Multivariable (BW-WALD) OR (%95 CI)	p
**Gender**	**Female (ref)**	504 (47)						
	**Male**	555 (53)	1.24 (0.89–1.72)	0.199	1.34 (0.95–1.88)	0.09		
**Hypertension**	**No (ref)**	340 (32)						
	**Yes**	719 (68)	1.78 (1.20–2.64)	0.004*	1.64 (1.08–2.48)	0.019*	1.74 (1.19–2.55)	0.004*
**DM**	**No (ref)**	276 (26)						
	**Yes**	783 (74)	1.12 (0.81–1.54)	0.49	0.92 (0.62–1.35)	0.65		
**NIHSS**	**1 (ref)**	425 (40)						
	**2**	549 (52)	0.98 (0.69–1.38)	0.89	1.05 (0.73–1.49)	0.79		
	**3**	85 (8)	0.96 (0.52–1.79)	0.91	0.98 (0.52–1.87)	0.96		
**PSD vs Non-depressive patients**	**Non-depressives (ref)**	679 (78)						
	**PSD**	187 (22)	1.70 (1.17–2.47)	0.005*	1.71 (1.17–2.51)	0.006*	1.64 (1.15–2.35)	0.007*
**Smoking**	**No (ref)**	769 (73)						
	**Yes**	290 (27)	0.79 (0.55–1.16)	0.24	0.931 (0.63–1.38)	0.72		
**Atrial fibrillation**	**No (ref)**	813 (77)						
	**Yes**	246 (23)	1.77 (1.23–2.55)	0.002*	1.72 (1.18–2.49)	0.004*	1.61 (1.14–2.28)	0.007*
**Hyperlipidemia**	**No (ref)**	439 (42)						
	**Yes**	620 (58)	1.04 (0.75–1.46)	0.80	1.06 (0.74–1.51)	0.75		
**Education**			1.01 (0.96–1.07)	0.61	1.02 (0.96–1.07)	0.54		
**Age**			1.03 (1.01–1.04)	<0.001*	1.02 (1.01–1.04)	0.007*	1.02 (1.00–1.03)	0.019*

Analysis included univariate, multivariable, and backward Wald regression. Results are shown as odds ratios (OR) with 95% confidence intervals (CI).

*DM* diabetes mellitus; *NIHSS* the national institutes of health stroke scale; *N* number of cases; *PSD* poststroke depressive patients; *Ref* reference category.

*p < 0.05 was considered statistically significant.

Second, all variables were entered together into a multivariable logistic regression model to evaluate their contributions to stroke recurrence. Finally, using the backward Wald method, the last model retained four significant predictors: poststroke depression (p = 0.007; OR = 1.64; CI 1.15–2.35), hypertension (p = 0.004; OR = 1.74; CI 1.19–2.55), atrial fibrillation (p = 0.007; OR = 1.61; CI 1.14–2.28), and older age (p = 0.019; OR = 1.02; CI 1.003–1.03). These variables emerged as the main independent risk factors for stroke recurrence at 12 months. In contrast, other factors (gender, diabetes mellitus, smoking, hyperlipidemia, education, and NIHSS score) were not significant in the final model (Table 5, Fig. 3).

## Discussion

The overall findings of this study are summarised across three main domains: the impact of stroke location on depression, the influence of post-stroke depression on cognitive impairment, and the effect of post-stroke depression on stroke recurrence.

In terms of poststroke depression, we observed a higher rate of depression associated with lesions affecting the cortico-subcortical structures in patients receiving no antidepressant treatment in this study, compared to stroke patients without depression. The involvement of cortico-subcortical structures was unsurprising, since these appear to contribute to the development of depression, particularly by affecting the frontal white matter tracts and subcortical structures involved in mood regulation [27]. The role of the location of stroke in the development of post-stroke depression and cognitive impairment has been suggested in a previous study by Jun Tu et al, although this study emphasised that anterior circulation strokes were more commonly associated with PSD development compared to posterior circulation strokes and lacunar infarcts [28].

As mentioned briefly in the introduction section, this is consistent with previous research suggesting the role of cortico-subcortical connections, rather than separate regions, in the development of PSD. This is consistent with the recently proposed vascular depression hypothesis, indicating a bidirectional relationship between stroke and depression [4, 18]. For instance, previous studies have shown that ischemic changes, particularly in the left-sided cortical and subcortical (white matter) structures, affect regions of the brain that are important for cognition and emotions [29, 30].

Regarding cognition, we also observed impaired cognitive scores in patients with PSD compared to those with pure stroke. Also, a deeper investigation of this finding revealed that a diagnosis of depression independently increased the risk of impairment in executive functions (OR = 4.5), episodic memory (OR = 4.65), semantic memory (OR = 4.79), language (OR = 5.09), and visuospatial deficiencies (OR = 6.1). This again aligns with previous studies [14, 31], including one of our own [32], showing the detrimental role of PSD in cognitive impairment. In that context, several studies have shown that post-stroke depression is not only linked to impaired cognition but also associated with an increased risk of developing dementia in stroke patients [15]. Additionally, from this perspective, our results agree with previous findings showing that depression and PSD both may contribute to a broad range of cognitive disorders, although it is explicitly difficult to elucidate the mechanistic link between stroke and depression and eliminate the underlying bidirectional pathophysiological link between these disease conditions, particularly when considering the vascular risk factors responsible for developing both disorders [16, 17]. Nevertheless, our findings are consistent with previous research showing that 10 mg/day citalopram antidepressant treatment significantly reduces stroke recurrence in a population of 440 individuals [20].

Furthermore, our finding of an increased rate of stroke recurrence in PSD in 52 weeks is worth discussing. This is in line with several studies confirming the role of PSD in increased stroke mortality and stroke recurrence [33–35]. These studies cumulatively indicated that not only post-stroke depression, but also depressive symptoms observed after stroke might increase stroke mortality and recurrence [33–35].

However, the difficulty in determining precisely whether depression itself is an independent risk factor for stroke recurrence or exhibits multiple vascular risk factors that may be responsible for increased stroke recurrence is also deserving of mention. For instance, even though the majority of our PSD patients exhibited multiple vascular risk factors similar to those in non-depressive stroke patients (Chi-Square test, p > 0.05, Table 2), stroke recurrency was more observed in PSD. The role of depression on stroke recurrence was also suggested by our univariate analysis, which identified depression, as an independent risk factor of stroke recurrence. These results align with previous studies identifying depression as an independent risk factor for stroke events [36, 37]. In line with several studies proposing that depression may contribute to stroke via different mechanisms. These mechanisms include its direct effects on neuroendocrine system (e.g., sympathetic nervous system activation, dysregulation of the hypothalamic-pituitary-adrenocortical axis, platelet aggregation dysfunction) immunological/inflammation effects [3, 38], as well as its indirect association with poor health behaviors (i.e., smoking, physical inactivity, poor diet, lack of medication compliance) [39], and obesity [40].

A potential limitation of this study is that the patients who participated may have had different behavioral, psychological, and physical characteristics from those in a population-based stroke cohort, and we have not evaluated the quality-of-life index of stroke patients in our present study. In addition, this study also did not include a healthy comparison group, making the investigation of the independent effect of depression on vascular risk factors in patients with PSD challenging compared to healthy individuals. However, a previous study of stroke and depression has not included detailed assessments of neuropsychological variables and has employed different methodologies, by omitting to recruit representative samples, adopting cross-sectional designs, and involving relatively small sample sizes [12].

Despite these minor limitations, it is worth noting that our results are valuable in terms of their structural nature. For instance, a recent study by Pan et al. [41] demonstrated that structural disconnection (SDC) was better than indirect functional connectivity measures for predicting post-stroke depression, and provided strong evidence that SDC across multiple brain regions independently contributes to PSD. Relevant to our study, the authors specifically determined that disconnections between bilateral limbic and prefrontal regions were strongly associated with PSD that supported both fronto-limbic model and vascular depression hypothesis. These findings, together with our results, suggest that PSD is not only a psychosocial response to stroke but also occurs directly from neurobiological injury, and is concordant with depression’s biopsychosocial model. Our findings could be important in clinical neurology and psychiatry practice, emphasising not only the role of depression as an independent risk factor for stroke recurrence but also the specific stroke location for the development of post-stroke depression, both of which might help us develop preventive therapeutic strategies against stroke recurrence and depression after stroke.

## Conclusion

To summarise, the present study provides strong evidence confirming the importance of depression in predicting cognitive impairment and recurrence in stroke patients. Nevertheless, our findings warrant the performance of further research to demonstrate the efficacy of treatment on stroke recurrence, together with other vascular risk factors and cognitive disorders. Future research should explore potential interventions targeting these multifaceted pathways, integrating both pharmacological and psychosocial strategies to mitigate the impact of depression in stroke rehabilitation.

## Data Availability

The datasets used and/or analysed during the current study are available from the corresponding author on reasonable request due to ethical reasons.
